# Ticks and Tularemia: Do We Know What We Don't Know?

**DOI:** 10.3389/fcimb.2019.00146

**Published:** 2019-05-08

**Authors:** Briana Zellner, Jason F. Huntley

**Affiliations:** Department of Medical Microbiology and Immunology, University of Toledo College of Medicine and Life Sciences, Toledo, OH, United States

**Keywords:** tularemia, tick, *Francisella tularensis*, vector-borne disease, *Dermacentor*, *Amblyomma*

## Abstract

*Francisella tularensis*, the causative agent of the zoonotic disease tularemia, is characterized by high morbidity and mortality rates in over 190 different mammalian species, including humans. Based on its low infectious dose, multiple routes of infection, and ability to induce rapid and lethal disease, *F. tularensis* has been recognized as a severe public health threat—being designated as a NIH Category A Priority Pathogen and a CDC Tier 1 Select Agent. Despite concerns over its use as a bioweapon, most U.S. tularemia cases are tick-mediated and ticks are believed to be the major environmental reservoir for *F. tularensis* in the U.S. The American dog tick (*Dermacentor variabilis*) has been reported to be the primary tick vector for *F. tularensis*, but the lone star tick (*Amblyomma americanum*) and other tick species also have been shown to harbor *F. tularensis*. This review highlights what is known, not known, and is debated, about the roles of different tick species as environmental reservoirs and transmission vectors for a variety of *F. tularensis* genotypes/strains.

## Introduction

*Francisella tularensis* (*Ft*), the causative agent of the zoonotic disease tularemia, can infect and cause lethal disease in over 300 species, including humans (Dennis et al., 2001; Keim et al., 2007). This Gram-negative coccobacillus is divided into three subspecies: subsp. *tularensis* (Type A), subsp. *holarctica* (Type B), and subsp. *mediasiatica*. However, only subsp. *tularensis* and subsp. *holarctica* are virulent for humans. A separate species, *F. novicida*, is associated with rare disease in immunocompromised humans and is sometimes used as a surrogate to study *Ft* pathogenesis (Oyston and Quarry, 2005; Kingry and Petersen, 2014). Type A strains, found solely in North America, are the most virulent for humans with a low infectious dose (<10 organisms) and high mortality rates (up to 60% mortality if untreated) (Ellis et al., 2002). Type B strains, although less virulent, still cause debilitating illness and are distributed throughout the northern hemisphere (Ellis et al., 2002; Oyston and Quarry, 2005). Type A strains can be further divided into three subpopulations: A1a, A1b, and A2, with A1b causing the most serious infections (Kugeler et al., 2009). Interest in tularemia research has increased over the past two decades due to the classification of this organism as a Tier 1 select agent by the U.S. Centers for Disease Control, highlighting the high morbidity and mortality, ease of aerosolization, and low infectious dose of this pathogen (Petersen and Schriefer, 2005). Aside from aerosolization, *Ft* can be transmitted to humans via the handling of infected animal carcasses, ingestion of contaminated food or water, or by bites by infected arthropods (Petersen et al., 2009).

**Figure 1:**
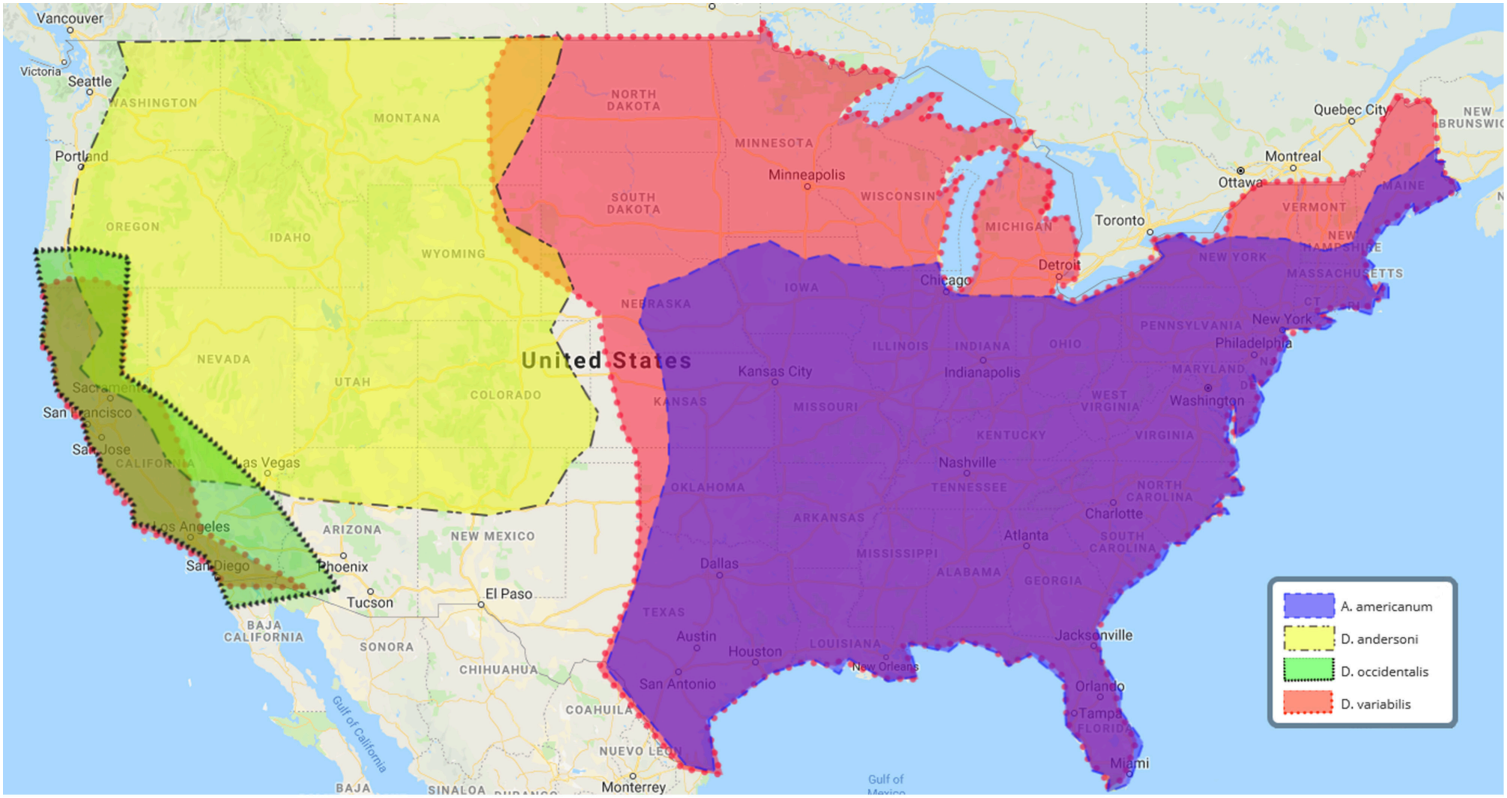
U.S. geographic distribution of ticks associated with human tularemia. Data adapted from the Centers for Disease Control and Prevention, https://www.cdc.gov/ticks/geographic__distribution.html.

In the U.S. alone, tick-borne disease (TBD) cases have nearly doubled between 2004 and 2016, with nearly 50,000 TBD reported in 2016. TBD include Lyme disease, anaplasmosis/ehrlichiosis, spotted fever, babesiosis, Powassan virus, and tularemia (Rosenberg et al., 2018). Ticks initially were discovered as a vector of tularemia in 1923 (Parker et al., 1924). In the 1960's, 85% of all tularemia cases in the south-central U.S. were reported to be associated with tick exposure (Brooks and Buchanan, 1970). More recently, approximately half of U.S. tularemia infections are tick-associated (Eisen, 2007; Rosenberg et al., 2018). Ulceroglandular tularemia, the most common presentation of the disease in the U.S., typically is attributed to bites by infected arthropods (Ellis et al., 2002). In the U.S., the most commonly reported tularemia tick vectors include *Amblyomma americanum, Dermacentor andersoni, D. occidentalis*, and *Dermacentor variabilis* (Figure 1 and Table 1). In Europe, *D. reticulatus* and *Ixodes ricinus* are most frequently associated with *Ft* (Table 1). These ticks are members of the family Ixodidae (hard ticks) but variations in their host preference, geographic distribution, and habitat likely influence their ability to transmit *Ft* (Table 1). Despite evidence that ticks are important for both the environmental persistence and transmission of *Ft* (Goethert and Telford, 2010), major questions remain about which tick species allow *Ft* replication and persistence, transmit *Ft* to naïve hosts, or prime *Ft* for mammalian infection. A cursory review of published literature indicates that despite over 1,300 reports of *Ft* infections in humans and animals, <10% (*n* = 141) of those examined the role of ticks—highlighting that *Ft*-tick studies are understudied. This review will highlight what is known, and not known, about *Ft* prevalence in different ticks, *Ft* transmission by infected ticks, *Ft*-tick interactions, and areas for future research.

**Table 1:** Ticks Associated with Human Tularemia.

**Tick species**	**Host preference^a^**	**Preferred habitat**	**Transmit *Ft*?^b^**	***Ft s*ubspecies^C^**	**Transovarial transmission?^C^**	**Transstadial transmission?^C^**	**References**
*Amblyomma americanum* (lone star tick)	Humans (L,N,A); small&large animals (N&L); large animals (A)	Woodlands	Yes (Exp&Nat)	Type B	No	Yes	(Calhoun, 1954; Mani et al., 2015; Sonenshine, 2018; Raghavan et al., 2019)
*Dermacentor andersoni* (Rocky Mountain wood tick)	Rodents, rarely humans (L&N), large mammals, humans (A)	Shrubs, tall grasses, and lightly wooded areas	Yes (Exp&Nat)	Type A	Yes	Yes	(Parker et al., 1924); (Mather, 2005; Sonenshine, 2018)
*Dermacentor occidentalis* (Pacific coast tick)	Small rodents and mammals (L&N), humans (N&A); large animals (A)	Shrubs	Yes (Nat)	Unk	Unk	Unk	(Parker et al., 1929); (Mather, 2005)
*Dermacentor reticulatus* (ornate cow tick)	Small mammals (L&N); medium mammals, sometimes humans (N); medium-large mammals, humans (A)	River basins, vegetation	Yes (Nat)	Type B	No	Unk	(Genchi et al., 2015; Földvári et al., 2016)
*Dermacentor variabilis* (American dog tick)	Host-specific (N&L); small mammals (L&N); small-medium mammals, humans (A)	Vegetation, tall grasses	Yes (Exp&Nat)	Type A&B	No	Yes	(Goethert et al., 2004; Mather, 2005; Mani et al., 2012; Sonenshine, 2018)
*Ixodes ricinus* (castor bean tick)	Humans (L,N,A); small-medium animals, mostly rodents (L); mostly birds and rodents (N); large animals (A)	Shrubs, tall grasses, deciduous woodlands	Yes (Nat)	Type B	No	Unk	(Genchi et al., 2015; Sonenshine, 2018; Sprong et al., 2018; Wilson and Elston, 2018)

a L, larvae; N, nymph; A, adult.

b Exp, experimental; Nat, natural.

c Unk, unknown.

### Tularemia-Associated Tick Species, Tick Infection Rates, and Geographic Locations

From 2004 to 2016, 2,102 tick-borne tularemia cases were reported to the U.S. National Notifiable Disease Surveillance System (Rosenberg et al., 2018), with the majority of infections occurring in Missouri and Arkansas (Eisen, 2007). *D. variabilis* (American dog tick) and *A. americanum* (lone star tick), arguably the two most important tick vectors of U.S. human tularemia, both are found in Missouri and Arkansas (Figure 1) (Petersen et al., 2009). Seasonal peaks of tularemia, April–August, correlate with the active period for both tick species (Eisen, 2007). *D. variabilis* has the widest geographic range, being found in nearly every state east of the Rocky Mountains and most of California (Figure 1). By comparison, *A. americanum* is confined mainly to the south-east U.S. (Figure 1). Although both *D. variabilis* and *A. americanum* are naturally infected with *Ft* (Calhoun, 1954; Goethert et al., 2004), percentages of ticks infected by Type A or Type B *Ft* are unknown. At least three studies have demonstrated that *A. americanum* and *D. variabilis* can maintain *Ft* infections over winter (or for >4 months), supporting their role as environmental reservoirs (Hopla, 1953, 1960;Mani et al., 2015).

Ticks are responsible for the majority of U.S. tularemia cases, yet *Ft*-tick prevalence studies indicate wide variations of infected ticks in the environment: < 0.1% of Minnesota *D. variabilis* ticks (*n* = 2,000) were *Ft*-infected (Green, 1931)*;* 17% of South Dakota *D. variabilis* ticks were *Ft*-infected (Markowitz et al., 1985); no Mississippi *A. americanum* ticks (*n* = 191) were *Ft*-infected (Castellaw et al., 2010); finally, 2% of Arkansas *A. americanum* ticks (*n* = 12,845) were *Ft*-infected but no Arkansas *D. variabilis* (*n* = 2,201) *Haemaphysalis leporispalustris* (rabbit tick; *n* = 1,494) or *Ixodes scapularis* (deer/blacklegged tick; vector for Lyme disease and many other pathogens; *n* = 142) were *Ft*-infected (Calhoun, 1954).

Martha's Vineyard, Massachusetts is an important site in the epidemiology of U.S. tularemia, as two major outbreaks have been reported: one in 1978 affecting 15 people and a second in 2000 affecting 15 people (Goethert et al., 2004). Although the cause of each outbreak remains unknown, four of the cases were linked to bites from *D. variabilis* ticks (Goethert et al., 2004). Analysis of >4,200 Martha's Vineyard *D. variabilis* ticks following the 2000 outbreak revealed that 0.7% were infected with Type A *Ft* but no other ticks (*Ixodes dammini* deer ticks; >600 tested) were infected (Goethert et al., 2004). Although sequence analyses of *fopA* (outer membrane protein) and PPI-helicase from these *Ft* strains indicated that they were nearly identical to the Type A reference strain SchuS4, multiple tandem-repeat analysis of two loci identified 10 unique genotypes, indicating that the degree of *Ft* genetic diversity on Martha's Vineyard is as great as the diversity found in *Ft* strains across North America and that Martha's Vineyard has a long history of enzootic *Ft* transmission (Goethert et al., 2004). Between 2004 and 2007, *Ft* DNA was detected in 2.7–4.3% of Martha's Vineyard *D. variabilis* ticks (>7,000 ticks tested), with 13 different *Ft* genotypes being identified by multiple tandem-repeat analysis (Goethert and Telford, 2009). Importantly, *Ft* numbers in Martha's Vineyard infected ticks were found to range from 0 to 10^11^ *Ft* genome equivalents (ge)/tick, with half of ticks harboring 10^8^-10^9^ *Ft* ge/tick (Goethert and Telford, 2010).

Dogs have been implicated to bring infected ticks into contact with humans. Early studies reported that *Ft* was detected in 0.4% of *A. americanum* ticks collected from Arkansas dogs (Calhoun, 1954). From 2006 to 2016, 1,814 U.S. human tularemia cases were reported, 735 (40%) of which had records indicating how exposure might have occurred (Kwit et al., 2018). Of those, 24 (3.3%) were dog-related and four (0.5%) were due to tick exposure from dogs (Kwit et al., 2018). In 1984, a tick-borne tularemia outbreak in twenty people from South Dakota Indian reservations was linked to dog exposures, with 17% of *D. variabilis* ticks from dogs found to harbor either Type A (12.5%) or Type B (87.5%) *Ft* (Markowitz et al., 1985). Unfortunately, clinical isolates were not collected from those patients so correlations between transmission of Type A and Type B *Ft* from infected ticks could not be determined.

Rabbit and lagomorph infections likely have contributed to the perpetuation of tularemia in the environment and to humans. The rabbit tick, *H. leporispalustris*, which is distributed across North America, likely is important for transmitting *Ft* to rabbits (Hopla, 1960; Goethert and Telford, 2010) and has been found to be naturally infected with *Ft*. Those findings are in contrast to the previously referenced study that did not detect *Ft* in Arkansas *H. leporispalustris* ticks (Calhoun, 1954). Although *H. leporispalustris* was reported to transovarially transmit *Ft* to its offspring and serve as a reservoir for *F. tularensis* (Parker, 1934), *H. leporispalustris* has not been associated with human tularemia, questioning the relevance of *H. leporispalustris* to human disease. *Ft* also has been reported to naturally infect other ticks, including *Dermacentor andersoni* (Rocky Mountain wood tick; Figure 1) (Parker et al., 1924), *Dermacentor occidentalis* (Pacific coast tick; Figure 1) (Parker et al., 1929), and *Haemaphysalis cinnabarina* (bird tick) (Parker et al., 1932), but transmission of *Ft* from these ticks to humans needs further study.

*Ft-*infected ticks are not unique to the U.S., as *Ft* Type B has been found in several European tick vectors. Between 0 and 2.3% of *Dermacentor reticulatus* (ornate cow tick; *n* = 5,131; Table 1) in Austria, Czech Republic, Germany, Poland, and Slovakia were found to be infected with *Ft* Type B (Gurycová et al., 1995). *Ft* was not detected in *Ixodes ricinus* (castor bean tick; *n* = 8,994) in France, Denmark, Italy, the Netherlands, Norway, or Poland (Mancini et al., 2014; Michelet et al., 2014; Quarsten et al., 2015; Stensvold et al., 2015; Wójcik-Fatla et al., 2015). However, other studies noted that 0.02–3.8% (*n* = 123,761) of *I. ricinus* were *Ft* Type B infected in France, Germany, Poland, Serbia, Slovakia, and Switzerland (Table 1) (Gurycová et al., 1995; Milutinovic et al., 2008; Reis et al., 2011; Gehringer et al., 2013; Wójcik-Fatla et al., 2015; Tomaso et al., 2018; Wittwer et al., 2018). Finally, in Slovakia, 2.8% of *Haemaphysalis concinna* (bush tick; *n* = 35) were infected with *Ft* Type B (Gurycová et al., 1995). In summary, more information is needed about tick infection rates and infected tick species in the U.S., primarily in states with high tularemia rates (e.g., Arkansas, Colorado, Kansas, Missouri, Oklahoma, South Dakota). In addition, although more *Ft*-tick prevalence studies have been performed in Europe and more tularemia cases occur yearly in Europe (relative to the U.S.) (Hestvik et al., 2015), it still is unclear what tick species transmits *Ft* Type B in Europe or if differences in tick species and *Ft* genotypes between Europe and the U.S. correlate with differences in tularemia disease severity.

### *Francisella*-Like Endosymbionts

As noted above, ticks harbor and transmit several human pathogens but they also are colonized with endosymbionts that are closely related to pathogenic bacteria, offer fitness advantages to host ticks, and appear to promote pathogen acquisition/transmission (Bonnet et al., 2017). *Francisella*-like endosymbionts (FLEs) share 16s rDNA similarity to *Ft*, are widely distributed in many different ticks, replicate intracellularly, can be transmitted transovarially, and appear to have evolved from pathogenic *Ft* strains (Gerhart et al., 2016, 2018; Liu et al., 2016). However, unlike virulent *Ft*, FLEs do not grow in cell-free media and their transmission to and virulence in humans is unknown (Ivanov et al., 2011; Wójcik-Fatla et al., 2015). FLEs have been found in various *Dermacentor* sp., as well as *Hyalomma marginatum, Hyalomma aegyptium* (tortoise tick), and *Rhipicephalus sanguineus* (brown dog tick), among others (Ivanov et al., 2011; Wójcik-Fatla et al., 2015). Importantly, one U.S. study reported that up to 60% of ticks colonized with FLEs were falsely identified as *Ft*-positive when using 16S rRNA PCR only (Kugeler et al., 2005). However, additional testing of the same ticks using a *Ft* multitarget TaqMan assay, specifically amplifying the insertion sequence IS*Ftu2*, outer membrane lipoprotein *tul4*, and intracellular growth locus *iglC*, revealed that the ticks actually were not *Ft-*infected (Kugeler et al., 2005). The wide distribution of FLEs in different tick species is further highlighted by studies finding that >94% of *D. andersoni, D. variabilis*, and *D. occidentalis* ticks from the western U.S. were positive for FLEs (Niebylski et al., 1997; Rounds et al., 2012). A Canadian study reported that 86–93% of *D. variabilis* and *D. andersoni* ticks were colonized with FLEs (Dergousoff and Chilton, 2012). Further afield, 50% (*n* = 530) of Polish *D. reticulatus* ticks (Wójcik-Fatla et al., 2015), 84–100% (*n* = 257) of Israeli *Haemaphysalis* sp. ticks (Kreizinger et al., 2013; Azagi et al., 2017), and 3% (*n* = 361) of Hungarian *D. reticulatus* ticks have been found to contain FLEs (Kreizinger et al., 2013). FLEs are not the only microbe in ticks and, interestingly, FLEs were found to comprise up to 41% of the microbiome of California *D. occidentalis* ticks (no ticks were positive for *Ft*) (Gurfield et al., 2017). Another study reported that *Ft* and FLEs accounted for ~80% (20% *Ft*, 60% FLE) of the midgut microbiome of *D. andersoni* ticks collected in Oregon and Montana (Gall et al., 2016). In summary, because of genetic similarity to virulent *Ft*, FLEs may have artificially inflated *Ft* infection rates in some of the above referenced *Ft*-tick prevalence studies. In addition, although it is clear that FLEs are present in many ticks that transmit *Ft*, much more work is needed to determine if FLEs interact with *Ft*, determine if FLEs aid in *Ft* infection of ticks, and examine if FLEs play important roles in *Ft* transmission to naïve hosts.

### Transstadial Transmission of *F. tularensis* in Ticks

The tick lifecycle is complex, spanning up to 3 years, requiring a blood meal to transition from one life stage to the next (larva-nymph-adult), and requiring a final blood meal before mating and/or egg laying (Petersen et al., 2009). The frequency and length of tick blood meals depends on the type of tick (soft *vs*. hard) and on the tick species. Important for *Ft*, hard ticks (e.g., *A. americanum* and *D. variabilis*) feed for up to 11 days, taking two-thirds of the total blood volume in the last 24–48 h (Sojka et al., 2013). Female hard ticks feed once per life stage and die several days after oviposition. Because of this complex life cycle, there are questions about whether *Ft* can be transstadially-transmitted from one life stage to the next, if all tick life stages can transmit *Ft* to naïve hosts, or if infected female adult ticks can transovarially transmit *Ft* to their eggs.

*Ft*-infected *D. andersoni* and *D. variabilis* ticks have been shown to molt from larvae to nymphs and from nymphs to adults, demonstrating that transstadial transmission of *Ft* can occur at all life stages. Importantly, all tick life stages also were shown to transmit *Ft* to naive guinea pigs, hares, or rabbits (Parker et al., 1924; Philip and Jellison, 1934). More recent studies demonstrated the *Ft* Type B attenuated live vaccine strain (LVS) was transstadially-transmitted in *D. variabilis* larvae to nymphs and nymphs to adults, noting that bacterial numbers decreased before each molt, then increased 3–4 logs after each molt (Mani et al., 2012). However, only 22% of nymphs maintained LVS infection through day 28 post-infection (close to molting), 25% of those infected nymphs survived molting, and only 25% of LVS-infected adult ticks maintained LVS through day 165 post-infection. Because *D. variabilis* in those studies were artificially fed using capillary tubes, it is difficult to determine if most *Ft* infections are cleared in naturally-infected ticks or if natural *Ft* infections negatively impact molting (Mani et al., 2012). The authors of that study also capillary-fed *A. americanum* with LVS, observing transstadial transmission between all life stages, LVS decreases before molting, LVS increases after molting, and low maintenance of LVS over time (Mani et al., 2015). By comparison, one study noted very high transstadial transmission rates of virulent *Ft* strains from *D. variabilis* larvae to nymph (fed on infected mice): Type A1b (93.3%), Type A2 (96.7%), and Type B (100%) (Reese et al., 2010).

Although older studies detected *Ft* in the eggs of infected adult female *D. variabilis* ticks and noted that oviposition was unimpaired by infected ticks (Bell, 1945), neither study examined if *Ft* was present in hatched larvae. More recently, transovarial transmission from capillary-infected *A. americanum* or *D. variabilis* ticks was not observed (Mani et al., 2012, 2015). Additionally, while *Ft* Type B was detected in oocytes of infected adult female *D. reticulatus* and *I. ricinus* ticks fed on infected guinea pigs, transovarial transmission was not observed (Genchi et al., 2015). Taken together, it appears that *Ft* can be transstadially-transmitted between all tick life stages and all tick life stages have been reported to transmit *Ft* to naïve hosts. However, transovarial transmission of virulent *Ft* should be examined, more studies are needed to understand if naturally-infected ticks clear *Ft* over time, and additional studies are needed to examine transmission of virulent *Ft* by infected ticks to naive hosts.

### *F. tularensis-*Tick Interactions

Questions about potential negative impacts of *Ft* infections on ticks and whether ticks restrict *Ft* replication/persistence have been examined in a number of studies in *D. variabilis*. Whereas environmentally-collected ticks have a number of limitations (e.g., low infection rate), *Ft-*tick infection experiments in the laboratory have their own limitations, including targeting biologically-relevant *Ft* numbers in ticks, selecting the appropriate tick life stage to infect, and selecting the tick infection model (e.g., infected mouse *vs*. capillary feeding). These limitations are further confounded by ticks requiring 3–7 days to feed to repletion and mice succumbing to virulent *Ft* infection within 4–5 days (Coburn et al., 2015). Although blood meal feeding mimics natural infection cues, bacterial numbers can be highly variable (Hopla, 1953; Coburn et al., 2015) and some ticks die while feeding on an infected host, suggesting that *Ft* has negative impacts on ticks. Uninfected *D. variabilis* nymphs have been reported to survive significantly better (58.5% survival) than nymphs infected with *Ft* Type A2 (11.6% survival) or Type B (29.8% survival). Interestingly, no significant difference in survival of uninfected and Type A1b-infected *D. variabilis* was observed (Reese et al., 2010). In contrast, another study noted that A1b-infected adult *D. variabilis* ticks had significantly lower survival rates (82% survival) than uninfected (92% survival), A2-infected (95% survival), or Type B-infected ticks (90% survival) (Reese et al., 2011). *Ft* Type A1a also appears to negatively impact tick survival, as only 11% of A1a-infected *D. variabilis* collected from Martha's Vineyard survived 6 months, compared with 52% survival for uninfected ticks (Goethert and Telford, 2011). By comparison, an older study found no significant difference in mortality rates between uninfected and *Ft*-infected *D. variabilis* (Bell, 1945). Some evidence indicates that high bacterial numbers or rapid bacterial replication (2- to 5-log increases in *Ft* Type A2 over 65 days) in ticks correlated with tick mortality (Reese et al., 2010). In contrast, another study found virtually no difference in survival rates for *D. variabilis* that were either uninfected (65% survival) of capillary-infected with *Ft* LVS (63% survival) (Mani et al., 2012). Considering the wide variations in reported survival rates for both uninfected (52–92%) and *Ft-*infected ticks (11% to 95% survival), it is difficult to conclude if *Ft* infections negatively impact ticks or if these results hold true for other tick species, including *A. americanum*.

With respect to *Ft* numbers and replication in ticks, two studies reported that *Ft* LVS numbers decline in capillary-tube fed *D. variabilis* or *A. americanum* ticks (Mani et al., 2012, 2015). For naturally-infected ticks, it has been speculated that anti-*Ft* antibodies from the mammalian host may limit bacterial replication/survival in ticks, as *D. variabilis* ticks fed on an immune host cleared *Ft* infections (Bell, 1945). Conversely, it also has been reported that *Ft*-infected *A. americanum* nymphs fed on hyperimmune dogs, rabbits, or rats retained *Ft* infections (Hopla, 1953, 1960). A fairly recent study reported reproducible tick infections by placing *D. variabilis* nymphs onto uninfected mice for approx. 77 h, retro-orbitally infecting those mice with 10^6^−10^8^ CFU of *Ft* LVS, and harvesting ticks 24 h later. In that study, mouse blood CFU/ml directly correlated with CFU/tick, *Ft* numbers increased over time in *D. variabilis* (after an initial decrease), and *Ft* doses <10^6^ CFU resulted in less efficient infection of and maintenance in ticks through molting to adult, indicating that a threshold of *F. tularensis* is needed to infect *D. variabilis* (Coburn et al., 2015). Similarly, another study noted that ticks must feed on an infected host during peak bacteremia to become infected (Bell, 1945). Finally, another study concluded that, as compared to direct injections of *Ft*, natural infections of ticks (feeding on an infected host) are necessary for proper colonization and bacterial dissemination (Genchi et al., 2015).

In theory, capillary tube feeding or direct injection of *Ft* into ticks can produce more consistent, standardized infections, but these methods lack natural infection cues (Mani et al., 2012, 2015). In one capillary feeding study, *D. variabilis* nymphs were capillary fed 10^7^ CFU/ml *Ft* LVS. One day later, only 30% of nymphs were infected and, of those, bacterial numbers were 4-logs less than the infectious dose (Mani et al., 2012). In direct injection studies, < 2 CFU *Ft* LVS delivered into the hemocoel of *D. variabilis* adults resulted in ~40% infection rate, whereas similar *A. americanum* adult injections did not establish infections (Mani et al., 2012, 2015).

Compared to *Borrelia burgdorferi*, which is found exclusively in *I. scapularis* midguts (De Silva and Fikrig, 1995), *Ft* has been reported to quickly (<24 h) disseminate from the gut to hemolymph and salivary glands of capillary-fed *A. americanum* ticks (Mani et al., 2015). *Ft* dissemination is further supported by one study noting that *Ft* migrated to the salivary glands of *D. reticulatus* and *I. ricinus* 6 days after ticks were removed from infected guinea pigs (Genchi et al., 2015) and another study noting that capillary-fed *D. variabilis* maintained *Ft* in their guts for up to 21 days before the bacteria spread to hemolymph and salivary glands (Mani et al., 2012). Conversely, a separate study noted that *Ft* did not disseminate to *D. variabilis* salivary glands (Coburn et al., 2015).

Transmission efficiency of *Ft* from infected ticks to hosts appears to be dependent on many factors, including the *Ft* strain, tick species, tick attachment efficiency, and feeding time. Results from one study suggested that *Ft* infection decreases tick attachment rates to naïve mice, with 96% attachment for uninfected *D. variabilis* adults, 86% attachment for A1b-infected, 58% attachment for A2-infected, and 52% attachment for Type B-infected ticks. In addition, *Ft* infection appeared to limit tick feeding, with 46% of uninfected ticks feeding to repletion, and only 23% of A1b-infected ticks feeding to repletion (Reese et al., 2011). In another study, 55% of *D. variabilis* ticks on uninfected mice fed to repletion, compared with only 3.7% of ticks feeding to repletion on A2-infected mice, and most of the ticks dying while feeding on A1b- and A2-infected mice (Reese et al., 2010). Although those results indicate that *Ft* infections alter tick feeding behaviors, other variables could account for these findings, including the reported preference of adult *D. variabilis* ticks for larger hosts (Sonenshine, 2018). Interestingly, one study noted that different *Ft* genotypes may be transmitted to naïve hosts at different frequencies (using infected *D. variabilis*): Type A1b transmitted to 67% of mice; Type A2 transmitted to 89% of mice; and Type B transmitted to 58% of mice (Reese et al., 2011). Differences in transmission could not be correlated to differences in bacterial numbers in ticks, as bacterial burdens in A1b-infected ticks (>10^9^ CFU) were significantly higher than bacterial burdens in A2- or Type B-infected ticks (~10^8^ CFU) (Reese et al., 2011). Given these conflicting findings, more studies are needed to better understand if *Ft* infections negatively impact different tick species, if *Ft* infections alter tick feeding behaviors, and if *Ft* genotypes are transmitted to naïve hosts at different frequencies.

## Conclusions

A large number of complex studies have been performed to understand which tick vectors are infected with *Ft*, which ticks are most likely to transmit *Ft*, which *Ft* genotypes are most likely to be tick-transmitted, what tick life stage is the most infectious, or if *Ft* infections have impacts on ticks. The majority of *Ft*-tick studies have focused on *D. variabilis* which, in the U.S., has the widest geographic range (Figure 1) and is most often associated with human tularemia. The second major tick vector for U.S. tularemia appears to be *A. americanum*. However, a number of other ticks, including those that feed primarily on small mammals, likely play important roles in *Ft* environmental persistence (Table 1). FLEs are a relatively new research field and much remains to be learned about how they interact with virulent *Ft*, if they provide metabolites/nutrients that support *Ft* persistence/replication in ticks, or if they contribute to transmission and disease. All tick life stages appear to support *Ft* and *Ft* can be transstadially transmitted from larva-nymph-adult. However, more studies are needed to understand if naturally-infected ticks can control or restrict *Ft* persistence/replication or if *Ft* infections have negative consequences on infected ticks. Finally, although it is clear that *Ft* is transmitted from infected ticks to naïve hosts, detailed studies are needed to understand if *Ft* genotypes are transmitted at different efficiencies.

In many cases, it is difficult to directly compare the highlighted studies because of differences in tick infection techniques (e.g., feeding on infected animals, capillary tube feeding, intrahemocoelic injection), environmental *vs*. laboratory infections, animals that transmitted *Ft* to ticks (e.g., mice, guinea pigs, rabbits, dogs), tick life stage used (larvae, nymph, adult), *Ft* infectious dose, and *Ft* genotypes/strains used (A1a, A1b, A2, B, LVS). Given these differences, future studies should directly compare bacterial replication in different ticks over time, transstadial transmission efficiency in different ticks, survival rates of different infected ticks, and *Ft* transmission to naïve hosts for *D. variabilis* and *A. americanum*, as well as other relevant ticks.

Finally, very little is known about *Ft* genes/proteins required for tick infection, persistence/replication in ticks, and transmission to naïve hosts. To our knowledge, only one study investigated the ability of a *Ft* mutant, a Δ*purMCD* strain, to infect and replicate in ticks (Coburn et al., 2015). Although Δ*purMCD* is avirulent in mice, it successfully colonized *D. variabilis* but was unable to persist in these ticks through the molt to the adult stage (Coburn et al., 2015). This finding indicated that, similar to biosynthetic pathways required for mammalian infections, the ability of *Ft* to synthesize purines is essential for replication in ticks. Studies to identify *Ft* genes/proteins required for persistence/replication in ticks, or the development of small molecule inhibitors that block *Ft* persistence/replication in ticks, could be important for reducing bacterial numbers in the environment, limiting enzootic episodes, and reducing human tularemia infections.

## Author Contributions

BZ and JH both read and reviewed all referenced papers and wrote this review.

### Conflict of Interest Statement

The authors declare that the research was conducted in the absence of any commercial or financial relationships that could be construed as a potential conflict of interest.
